# Modulation of Reaction Times and Sense of Agency via Subliminal Priming in Functional Movement Disorders

**DOI:** 10.3389/fneur.2020.00989

**Published:** 2020-09-11

**Authors:** Anne-Catherine M. L. Huys, Mark J. Edwards, Kailash P. Bhatia, Patrick Haggard

**Affiliations:** ^1^Department of Clinical and Movement Neurosciences, University College London Queen Square Institute of Neurology, London, United Kingdom; ^2^Neuroscience Research Centre, Institute of Molecular and Cell Sciences, St George's University of London, London, United Kingdom; ^3^Institute of Cognitive Neuroscience, University College London, London, United Kingdom

**Keywords:** subliminal priming, supraliminal priming, agency, functional movement disorders, functional neurological disorder, conversion disorder, motor control

## Abstract

**Background:** In functional movement disorders, explicit movements are impaired, while implicit movements are preserved. Furthermore, there is evidence that the sense of agency is abnormal.

**Aim:** We aimed to investigate how motor responses and sense of agency were affected by subliminal or supraliminal cues in people with functional movement disorders.

**Methods:** Twenty-three people with a functional movement disorder and 26 healthy controls took part in a subliminal and supraliminal priming experiment which investigated reaction times, choice and sense of agency. Participants pressed a left or right arrow key in response to an imperative left or right pointing arrow. Either key could be pressed in response to bidirectional arrows. The imperative arrow was preceded by a small left or right pointing prime arrow, that was non-predictive (50% correct) and was presented in either subliminal or supraliminal conditions. The participant's response caused the appearance of a colored circle and they rated the degree of control they felt over its appearance (sense of agency). The circle's color depended on whether their response was congruent or incongruent with the prime arrow direction. After exclusion, 19 participants remained in each group.

**Results:** Prime-compatible responses led to faster reaction times in both the subliminal and supraliminal condition. Subliminal prime-compatible responses were chosen more frequently in the free choice condition. The sense of agency did not depend on prime-response congruency. There were no significant differences in any of these measures between the two groups.

**Conclusion:** With non-predictive cues, reaction times, choices, and the sense of agency remain normal in people with functional movement disorders, for both subliminal and supraliminal primes. The findings suggest that it is not so much conscious awareness of the movement, but rather conscious motor preparation that is detrimental to motor function in functional movement disorders.

## Introduction

Sense of agency refers to the sense of controlling one's own actions, and, through them, events in the outside world. In other words, it is the conscious experience that one has volitional or willed control over one's own actions and can therefore influence the environment (1).

There are several reasons for suspecting an abnormality of the sense of agency in functional movement disorders (FMD). FMD share many characteristics of voluntary movements: they manifest with attention and improve or disappear with distraction; voluntary movements interfere with them as exemplified by the phenomenon of “entrainment” (2); and some FMDs are preceded by a “Bereitschaftspotential” on electroencephalography, which is typically present before self-paced voluntary movements and absent in involuntary movements (3). Patients, however, clearly state that their abnormal movements are involuntary. Could it be that the abnormal movements in FMD are indeed voluntary movements in terms of the physiological pathway that generates them, but are anomalous in terms of the conscious experience that accompanies their generation? Indeed, previous studies, both of intentional binding (4) and of the experience of conscious intention (5), have indicated an abnormal sense of agency in FMD. Abnormal activation of the temporoparietal junction in FMD, an area commonly implicated in the sense of agency further points in this direction (6).

It remains an open question whether the sense of agency *per se* is abnormal in FMD or if it is only impaired in the context of volitional actions.

Another interesting aspect of FMD is that motor performance is generally normal with implicit movements, but impaired with explicit movements. Which aspect of explicit motor control leads to abnormal movements? We hypothesized that increasing the attention and awareness associated with a movement would impair motor performance and sense of agency, in an FMD group, though not in healthy volunteers.

Subliminal priming offers a way of influencing actions or decisions, and thus influencing the voluntary motor pathway without the participant's awareness of this influence. A visual stimulus (the “prime”) is shown for a very short period, and its processing interrupted “masked” by the presentation of another stimulus (the “mask”) shortly after. The processing of the prime does thereby not reach consciousness, but it is nevertheless processed at a subliminal level thereby influencing subsequent responses. We suggest that subliminal priming therefore constitutes an implicit influence on the voluntary motor pathway, while supraliminal priming would explicitly influence the voluntary motor pathway.

Comparing the sense of agency with subliminal as opposed to supraliminal priming gives an opportunity to investigate whether it is the sense of agency *per se* that is affected in FMD, or if it is only affected in the context of conscious movement control. In addition, priming allows the measurement of further aspects of motor control, namely response speed and choice.

We therefore set out to evaluate, implicit vs. explicit motor control in terms of sense of agency, motor performance and action choice with the help of a subliminal and supraliminal priming paradigm.

## Methods

Twenty-three people with FMD and 26 age and gender matched healthy control subjects took part. Almost all patients were recruited from the clinical practice of experts in FMD (ME and KB), primarily as they were seen in inpatient or outpatient settings. People with any type of FMD were invited to participate, with the following exclusion criteria: presence of an organic neurological disorder (apart from headache disorders), cognitive impairment, inability to perform the experiment, and age under 18 or over 80. Several participants had previously taken part in a functional tremor study, which explains their relatively large proportion. All participants' diagnoses were confirmed by a further neurologist (ACH) on the day of the study, according to the diagnostic criteria described by Espay and Lang (7). She also ensured that the healthy controls did not have any undiagnosed movement disorder. The healthy control subjects were patients' family members, acquaintances, and healthy volunteers recruited from University College London's registries.

### Experimental Setup

**Figure 1:**
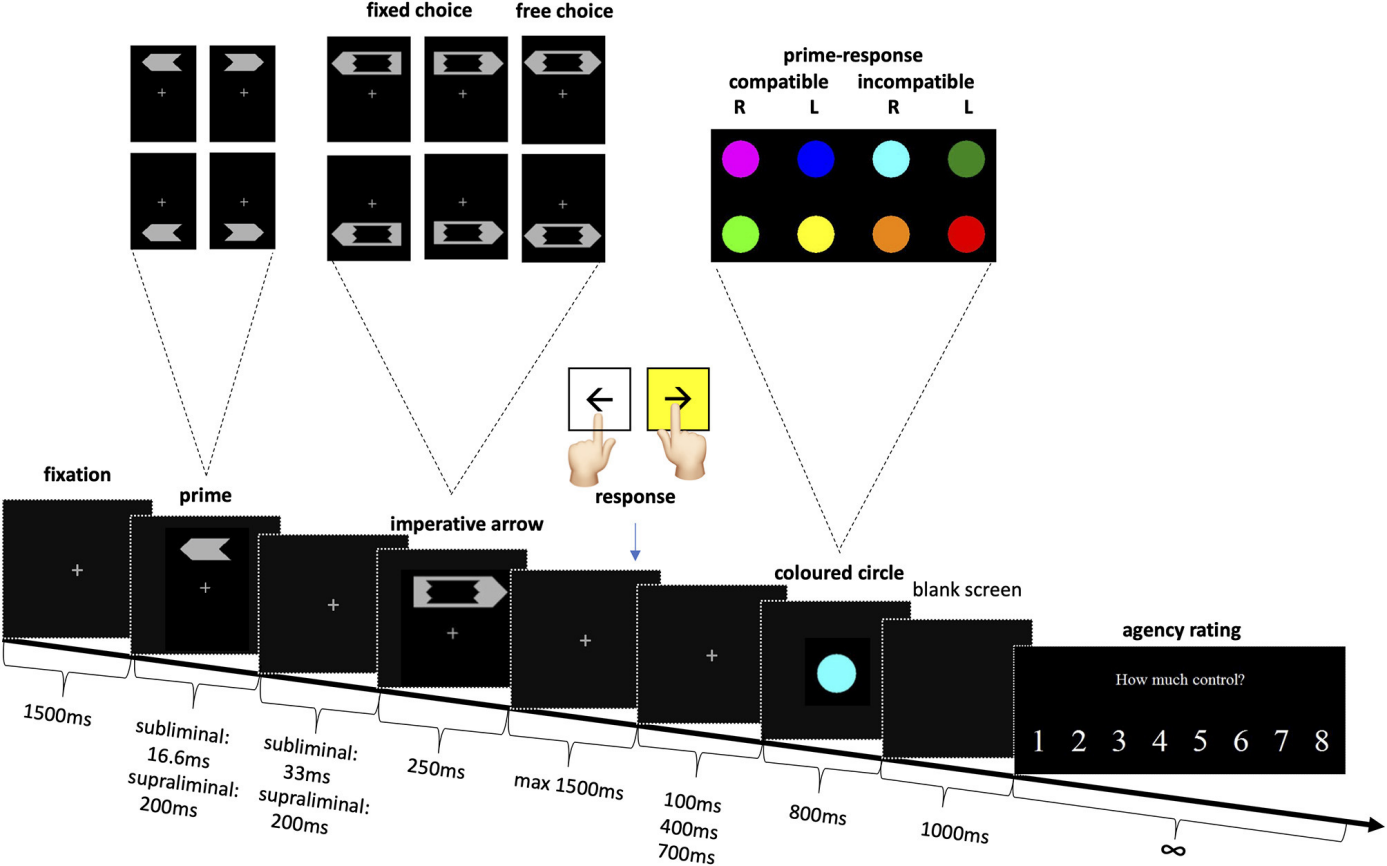
Experimental setup for subliminal and supraliminal priming with agency rating. Stimuli sizes in visual angle: prime height 0.8°, length 1.86°, mask height 1.09°, length 3.26°, circle diameter 1.86°.

The methods were adapted from earlier studies (8–10). Participants were seated, at a viewing distance of 65 cm, in front of a 19-inch computer screen on which the stimuli were presented. The task (Figure 1) was to press the corresponding left or right keyboard key as quickly as possible in response to a left or right pointing large imperative arrow (directional arrow “fixed choice”). In the case of a bidirectional imperative arrow (“free choice”), subjects could choose either key, but still had to do so as quickly as possible. They were encouraged to choose on the spot and not to follow a fixed pattern.

Before each large imperative arrow, a small prime arrow pointing either left or right was shown. In the subliminal condition the prime was presented for only 16.6 ms, i.e., subliminally and the subjects were not informed of its presence. In the supraliminal condition, the prime arrow was shown for 200 ms and participants were told to ignore it. The prime arrow and the imperative arrow, which also represented the mask, were isoluminant. So as to enhance the masking effect, both the prime, and the imperative arrow appeared randomly above or below the fixation point (8).

If the response was correct and sufficiently fast (maximum 1500 ms), a colored circle appeared. Unbeknown to the participants, the color of the circle reflected the congruence of their response to the prime. Eight colors were randomly allocated, so that two colors corresponded to right responses compatible to the prime; two colors to left prime compatible responses; two to right prime incompatible and the last two to left prime incompatible responses. After each trial, participants indicated how much control they felt they had over the appearance of the colored circle, on a scale from 1 (absolutely no control) to 8 (complete control).

Note that the interval between participant's response and the appearance of the colored circle was 100, 400, or 700 ms, so as to decrease predictability and hence lead to greater variability in the sense of agency. If the participant's response was too slow (>1500 ms) or incorrect in case of a directional arrow, a large “X” appeared on the screen. Incorrect trials were repeated up to four times at the end of the experiment, so as to avoid missing values.

All subliminal prime trials were performed separately from all supraliminal prime trials. Which one was performed first was assigned pseudo-randomly between participants. The participant was informed that the color attribution changed between the two conditions. The condition performed first contained 12 practice trials, the condition performed second contained two.

In the subliminal condition, there were 64 prime-fixed response compatible, 64 prime-fixed response incompatible and 64 free choice trials. Since the agency rating in the case of supraliminal primes is most relevant in the free choice condition, 120 free choice trials were presented, and only 30 prime-fixed response compatible and 30 prime-fixed response incompatible trials. Each condition was performed in six blocks, allowing for breaks in between. In the supraliminal condition, a short test at the start confirmed that participants were able to see both the small prime arrow and the larger imperative arrow. Stimuli were presented and the responses recorded using Matlab® R2015b (Mathworks, Natick, MA, USA) in conjunction with the Cogent 2000 toolbox (www.vislab.ucl.ac.uk/cogent.php).

### Exclusion

Participants who saw the supposedly subliminal prime had to be excluded. After the subliminal priming condition, participants were informed about the presence of the prime and asked to detect its direction in a prime visibility test. The settings were identical to those of the subliminal priming condition, with the exception of the absence of the colored circles and agency ratings. Furthermore, in order to prevent responses to the imperative arrow, participants could only respond 600 ms after the appearance of the imperative arrow, when the fixation cross turned green. If the person reported having sometimes seen the direction of the arrow prime, 120 trials were performed, otherwise 90. After the prime visibility test, participants were asked again if they had been able to see the direction of the prime arrow. Five healthy controls and two patients who either reported having seen the subliminal prime or who had a high discriminability index (d') in the prime visibility test were excluded. Two additional participants in both groups were excluded because they persistently gave the same agency rating. These participants were excluded prior to data being analyzed and replaced by new participants. The absence of a previous study of this task in FMD precluded meaningful sample size calculations. Instead, the sample sizes of 19 participants in each group were based on the sample sizes of the two previous subliminal priming studies on which the current one was based [21 participants in the healthy control study (9), 16 participants per group in the study comparing people with schizophrenia to healthy controls (10)]. It was similar to the sample size of 20 healthy controls in the agency study with supraliminal priming mentioned below (11). Furthermore, the sample size was relatively large, compared to previous reaction time studies in FMD with supraliminal premovement cues, which included 8 (12), 11 (13), and 21 (14) FMD patients. Thus, recruitment continued until there were 19 remaining participants in each group. Their characteristics are detailed in Table 1.

**Table 1:** Study participant characteristics.

	**FMD** **(*n =* 19)**	**Healthy control** **(*n =* 19)**
**M:F**	8:11	9:10
**Age:** average (*SD*) (range)	46.9 y (14.1) (20–64 y)	46.6 y (14.1) (32–79 y)
**FMD type**	Functional action tremor: 14 - upper limb: 14 - lower limb: 5 - head: 2 Functional weakness: 6 - upper limb: 2 - lower limb: 5 Functional dystonia: 4 - upper limb: 1 - lower limb: 1 - cervical: 1 - mandibular: 1 Functional gait disorder: 4 Paroxysmal FMD: 5 - Hyperkinetic: 5 - Hypokinetic: 2 Functional stiffness: 2 - lower limb: 2	none: 19

*Note that 12 patients had more than 1 type of FMD. All but 4 patients had an FMD of at least one upper limb. “Functional gait disorder” means that the gait disorder comprised a functional gait component not explained by any other listed FMD type. None of the paroxysmal FMD was present on the day of testing*.

Similar to a previous study (9), trials for which the reaction times fell more than 1.5 times the interquartile range above the 3rd or below the 1st quartile of the participant's responses in that specific condition (i.e., fixed choice, or free choice within the subliminal or supraliminal condition) were excluded as outliers from all analyses (Table 2).

**Table 2:** Percentage of trials excluded as outliers in both the subliminal and supraliminal conditions.

	**Subliminal**		**Supraliminal**	
	**Fixed choice**	**Free choice**	**Fixed choice**	**Free choice**
HC	5.5%	4.0%	3.4%	3.8%
FMD	5.0%	4.4%	5.2%	3.0%

### Analysis

Both the reaction time (RT) and the sense of agency data were analyzed by means of a mixed model ANOVA with group as the between-subject factor and prime-response congruence as the within-subject factor. The underlying assumptions, including sphericity, were checked and adjusted for whenever necessary. Since ANOVA is relatively robust to departures from normality (15) and the sample sizes were always equal, ANOVA was preferred, unless variances were markedly unequal. Effect size estimates were based on partial eta squared ($\eta_{\text{p}}^{2}$) measures. For the free choice trials, the *t-*test (or the non-parametric Wilcoxon signed rank test) with a hypothesized mean of 50%, was used to check if choices deviated from chance. A two-sample *t-*test (or the non-parametric Wilcoxon rank-sum test) tested if there was any difference in the percentage of prime-congruent free choices between the FMD and healthy control groups. The significance level for all tests was set at 0.05, two-tailed. Matlab® R2015b (Mathworks, Natick, MA, USA) and STATA® (StataCorp. 2013. Stata Statistical Software: Release 13. TX: StataCorp LP) were used for data analysis.

## Results

### Subliminal Priming

Our analyses investigated whether people with FMD perceived a higher sense of agency and had faster reaction times with compatible compared to incompatible primes; and when they had a choice if they chose prime compatible responses more frequently.

#### Agency

**Figure 2:**
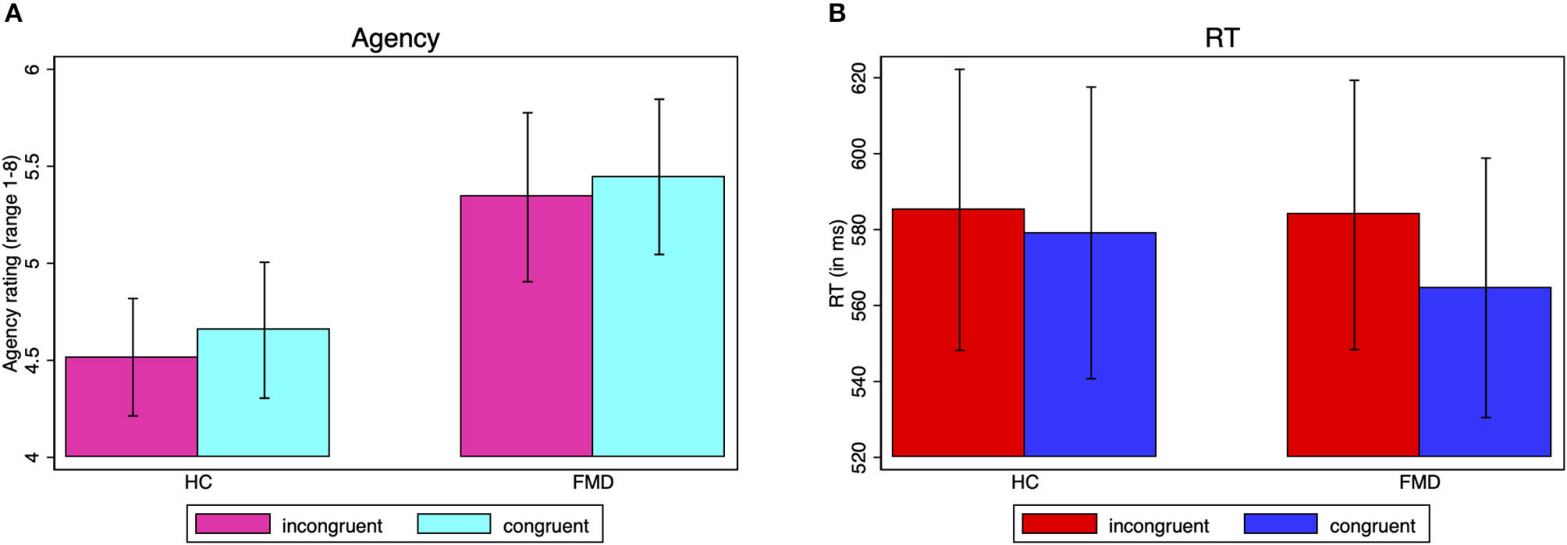
Subliminal priming agency ratings and reaction times. Average agency ratings **(A)** and reaction times **(B)** by group for prime-response incongruent vs. congruent responses, irrespective of the type of choice (unidirectional or bidirectional imperative arrow). The agency rating ranges from 1 (absolutely no control) to 8 (complete control). The standard error of the mean is shown by the error bars.

A mixed model ANOVA with group as between-subject factor and congruence as within-subject factor, with fixed and free choices collapsed together, indicated that the slight differences between the congruent and incongruent agency ratings seen in Figure 2A and Table 3 were not statistically significant (main effect of congruence [*F*_(1, 36)_ = 0.87, *p* = 0.36, $\eta_{\text{p}}^{2}$ = 0.024], nor was there a difference in the agency rating to prime congruent vs. incongruent responses between the groups (interaction group x congruence [*F*_(1, 36)_ = 0.02, *p* = 0.90, $\eta_{\text{p}}^{2}$ = 0.0005]. The main effect of group was not significant either [*F*_(1, 36)_ = 2.46, *p* = 0.13, $\eta_{\text{p}}^{2}$ = 0.064], and indeed would only have been a reflection of differences in the overall use of the scale.

**Table 3:** Agency rating group averages *(SD)* for different prime and choice conditions.

	**Subliminal prime**				**Supraliminal prime**			
	**Irrespective of choice mean**		**Free choice trials mean**		**Free choice trials mean**		**Fixed choice trials mean**	
	**Incongruent**	**Congruent**	**Incongruent**	**Congruent**	**Incongruent**	**Congruent**	**Incongruent**	**Congruent**
HC (*n =* 19)	4.52 (1.32)	4.66 (1.53)	4.52 (1.25)	4.66 (1.67)	4.65 (1.47)	4.92 (1.32)	4.65 (1.29)	4.87 (1.45)
FMD (*n =* 19)	5.34 (1.90)	5.45 (1.74)	5.32 (1.92)	5.43 (1.81)	5.48 (1.62)	5.44 (1.47)	5.50 (1.62)	5.68 (1.42)

*The table summarizes the group average agency ratings (standard deviations) according to the prime (subliminal or supraliminal prime, prime congruent, or prime incongruent responses) and the available choice: free choice (bidirectional imperative arrow); fixed choice (unidirectional imperative arrow); irrespective of choice (either uni- or bidirectional imperative arrow). The agency ratings range from 1 (absolutely no control) to 8 (complete control)*.

There is an argument to be made for perceiving a higher sense of agency with prime compatible as opposed to incompatible choices regardless of whether there is free choice or not. However, one could argue that the agency rating is most intuitively relevant in the case of free choices (bidirectional imperative arrows). Nevertheless, including only free choice responses (Table 3), the results were not significant for the main effect of congruence [*F*_(1, 36)_ = 0.62, *p* = 0.44, $\eta_{\text{p}}^{2}$ = 0.017] nor for the interaction group x congruence [*F*_(1, 36)_ = 0.01, *p* = 0.92, $\eta_{\text{p}}^{2}$ = 0.0003] either.

#### Reaction Time

A mixed model ANOVA with group as between-subject factor and congruence as within-subject factor, with fixed and free choices collapsed together, showed that the reaction times were significantly faster with compatible compared to incompatible primes, with a large effect size [main effect of congruence *F*_(1, 36)_ = 15.10, *p* = 0.0004, $\eta_{\text{p}}^{2}$ = 0.30] (Figure 2B). There was a trend for this effect to be more pronounced in the FMD group with a medium to large effect size (group x congruence interaction [*F*_(1, 36)_ = 4.08, *p* = 0.0509, $\eta_{\text{p}}^{2}$ = 0.10] (HC prime-response incongruent: *M* = 585.2 ms, *SD* = 161.4, congruent: *M* = 579.1 ms, *SD* = 167.5; FMD prime-response incongruent: *M* = 583.9 ms, *SD* = 154.6, congruent: *M* = 564.7 ms, *SD* = 148.9)). The main effect of group was not significant [*F*_(1, 36)_ = 0.02, *p* = 0.88, $\eta_{\text{p}}^{2}$ = 0.0007], indicating no significant difference in overall reaction times between the groups.

#### Choice

Significantly more prime congruent responses (53.7%) were chosen in free choice trials [Wilcoxon signed rank test with a hypothesized mean of 50% refuted the hypothesis that the choices were the same as chance (*Z* = 2.78, *p* = *0*.0055, *r* = 0.45)] and there was no significant difference between the two groups (HC 53.1%, FMD 54.4%; two-sample Wilcoxon rank-sum test *Z* = –0.70, *p* = *0*.48, *r* = –0.16).

### Supraliminal Priming

One questions of interest is whether people with FMD perceive a higher sense of control in free choice trials, when they choose the opposite of what has been suggested by the supraliminal prime, as has previously been shown in healthy controls (11).

The other relevant question is whether in fixed choice trials, compatible supraliminal primes lead to a stronger sense of agency and faster reaction times as opposed to incompatible supraliminal primes.

#### Agency

A mixed model ANOVA of the agency ratings in free choice trials (Table 3), with group as between-subject factor and congruence as within-subject factor did not give a significant main effect of congruence [*F*_(1, 36)_ = 0.68, *p* = 0.41, $\eta_{\text{p}}^{2}$ = 0.019] nor of the interaction group x congruence [*F*_(1, 36)_ = 1.30, *p* = 0.26, $\eta_{\text{p}}^{2}$ = 0.035].

Similarly, in fixed choice trials (Table 3), the main effect of congruence was not significant, [*F*_(1, 36)_ = 1.80, *p* = 0.19, $\eta_{\text{p}}^{2}$ = 0.048] nor was the interaction group x congruence [*F*_(1, 36)_ = 0.02, *p* = 0.88, $\eta_{\text{p}}^{2}$ = 0.0007].

#### Reaction Time

**Figure 3:**
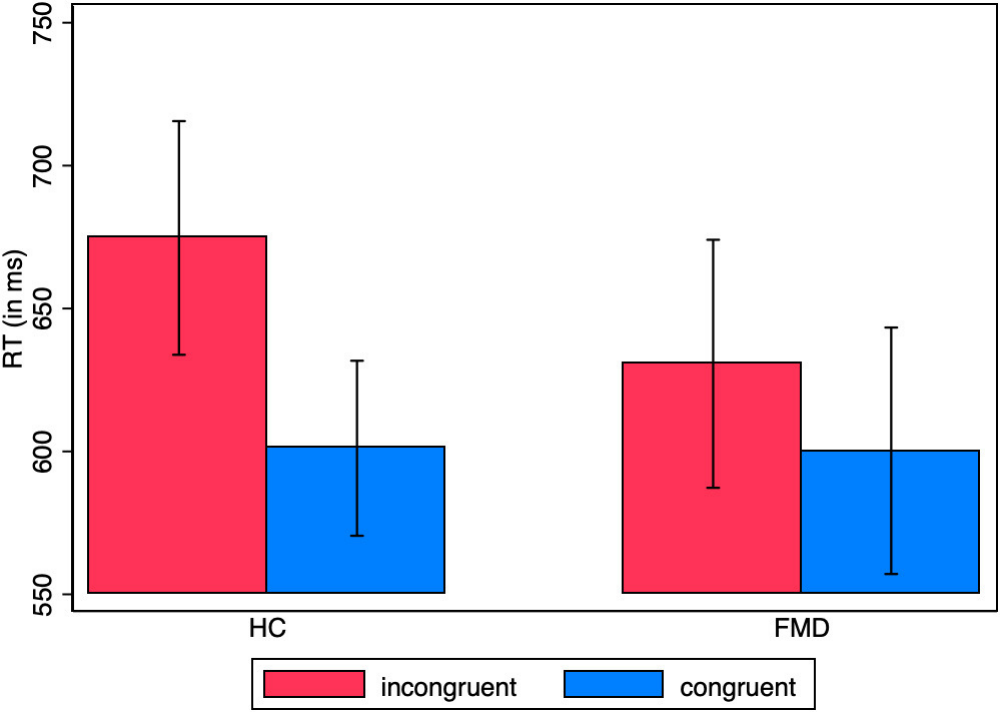
RT in supraliminal fixed choice trials. Average reaction times by group for prime-response congruent vs. incongruent responses, for fixed choice trials only (unidirectional imperative arrows). The standard error of the mean is shown by the error bars.

A mixed model ANOVA of the reaction times with group as between-subject factor and congruence as within-subject factor, for the fixed choices, gave a significant main effect of congruence with a large effect size [*F*_(1, 36)_ = 9.94, *p* = 0.0033, $\eta_{\text{p}}^{2}$ = 0.22] but not of group [*F*_(1, 36)_ = 0.17, *p* = 0.68, $\eta_{\text{p}}^{2}$ = 0.005], nor of the interaction group x congruence [*F*_(1, 36)_ = 1.71, *p* = 0.20, $\eta_{\text{p}}^{2}$ = 0.045] (HC prime-response incongruent: *M* = 674.7 ms, *SD* = 178.2, congruent: *M* = 601.1 ms, *SD* = 133.5; FMD prime-response incongruent: *M* = 630.7 ms, *SD* = 189.1, congruent: *M* = 600.2 ms, *SD* = 188.0) (Figure 3).

There was no systematic bias with regards to the chosen response, as there was no significant difference between the two groups (two-sample Wilcoxon rank sum test: *Z* = –0.75, *p* = 0.46, *r* = –0.17) and the chosen responses were not significantly different from chance (Wilcoxon signed rank test with a hypothesized mean of 50: *Z* = 1.62, *p* = 0.10, *r* = 0.26).

### Subliminal vs. Supraliminal Priming

Taking only free choice trials into account, there was no significant difference in agency ratings between the two groups according to whether the prime was presented subliminally or supraliminally [mixed model ANOVA with group as between-subject factor and prime type and congruence as within-subject factor, interaction effect group x prime: *F*_(1, 36)_ = 0.16, *p* = 0.70, $\eta_{\text{p}}^{2}$ = 0.0043], nor when prime-response congruence was also taken into account [group x prime x congruence interaction: *F*_(1, 36)_ = 0.56, *p* = 0.46, $\eta_{\text{p}}^{2}$ = 0.015].

Similarly, the reaction times in the fixed choice trials did not differ significantly between the two groups according to whether the prime was subliminal or supraliminal [mixed model ANOVA with group as between-subject factor and prime type and congruence as within-subject factor, prime x group interaction: *F*_(1, 36)_ = 0.09, *p* = 0.77, $\eta_{\text{p}}^{2}$ = 0.0025], nor when the prime-response congruence was also taken into account [prime x group x congruence interaction: *F*_(1, 36)_ = 2.16, *p* = 0.15, $\eta_{\text{p}}^{2}$ = 0.057].

### Side

In the nine people with asymmetric FMD symptoms of either the arm or the leg, the agency ratings and reaction times did not differ significantly between the more and less affected sides [agency ratings more affected side: *M* = 5.26, *SD* = 1.97; less affected side: *M* = 4.99, *SD* = 2.11; paired *t-*test: *t*_(8)_ = 1.15, *p* = 0.28; RT more affected side: *M* = 555.0 ms, *SD* = 145.8; less affected side: *M* = 559.0 ms, *SD* = 169.7; paired *t-*test: *t*_(8)_ = 0.348, *p* = 0.74].

## Discussion

To the best of our knowledge, this is the first subliminal priming study in FMD and indeed in functional neurological disorder in general. Subliminal priming was normal in terms of overall reaction times, modulation of reaction times with prime-congruence (faster responses with prime-congruent responses), influence on subsequent choices (more prime-congruent responses chosen in the free choice trials) and perceived sense of agency. These findings provide further support for normal implicit motor function in FMD.

A limitation of this study is the difficulty measuring how the sense of agency varies with minor changes in premovement priming. In this context, this study did not replicate findings of previous studies in which healthy controls felt more control with subliminal prime-response congruent as opposed to prime-response incongruent responses (9, 10). Although the effect in the present study pointed in the same direction, it did not reach statistical significance. A larger sample size might have led to a significant result, however, our sample size was similar to previous studies. Similarly, the present findings did not corroborate a previous study showing a higher sense of agency when choosing the opposite of what was suggested by the supraliminal prime (11). In the previous study a left or right button needed to be pressed as quickly as possible, once a row of crosses disappeared from the screen. The button press always generated a tone, but subjects were told that the tone might be generated by the computer. Subjects rated the degree they felt they rather than the computer had generated the tone. No primes, subliminal or supraliminal “LEFT” or “RIGHT” written primes were randomly intermixed. Our study paradigm was clearly different, involving fixed and free choices, different colors and no plausible external agent. These differences might therefore explain the non-replicability.

While people are generally aware of immediate outcomes of their actions, measuring varying degrees of agency under different experimental conditions is not straightforward. Participants may struggle to translate the everyday, implicit sense of agency into an unusual, explicit judgement. An implicit way of measuring the sense of agency is by means of the intentional binding paradigm: when an action is carried out in a voluntary fashion and followed by an effect (typically a button press, followed 250 ms later by a tone), then the perceived timing of this voluntary action and its effect move closer together (16). This temporal compression is a commonly accepted implicit proxy of the sense of agency. It could thus potentially replace the explicit agency rating in a future study. All in all, it is possible, that in the present study, the sense of agency measure was not sensitive enough to highlight possible differences.

A further possible limitation is that all but four patients in the present study, had an FMD affecting one or both their arms to some degree. Alternative ways of responding, such as a foot pedal or a voice switch could be used. Nevertheless, the study findings are very unlikely to be attributed to direct interference by the movement disorder as the responses in the FMD group did not differ from those of healthy controls, and there was no difference between the more and less symptomatic sides in the patients with asymmetric symptoms.

Despite these possible limitations, the conclusion of this study is that motor responses and the sense of agency are normal in FMD, both with subliminal and supraliminal priming. While normal subliminal priming was predicted, it is surprising that motor responses, in terms of reaction speed and sense of agency were normal with supraliminal priming. We had originally predicted that supraliminal priming would impair motor performance and sense of agency in the FMD group, though not in the control group, because supraliminal priming amounts to an explicit cue drawing attention to the impending movement—precisely the conditions where FMDs are most strongly manifested.

The most likely reason for these findings is that in FMD, predictability of actions and outcomes, and hence conscious motor experience, is key for abnormal movement to be expressed. Crucially, our primes were always non-predictive. Thus, any motor facilitation due to congruent vs. incongruent priming must reflect an *automatic* increase in preparation triggered by the congruent prime. Our results confirmed that this automatic motor facilitation did indeed occur in the FMD group, both for supraliminal and subliminal primes. In contrast, previous studies focussed on predictive, high-validity primes. Predictive primes allow a strategic, presumably conscious, preparation for the forthcoming movement. Two previous supraliminal priming studies showed faster reaction times with high cue predictability [95% valid as opposed to non-predictive (50%)] in healthy controls, but slower or unchanged reaction times with high cue predictability in FMD (13, 14). That is, whereas predictive supraliminal primes allow healthy volunteers to prepare movements in advance, this process did not operate as normal in FMD patients. Similarly, when a joystick movement could be prepared (because the location it needed to be moved to as quickly as possible once the go cue appeared was known in advance), FMD patients' movement times became gradually slower across the block (13). Thus, other studies have shown that patients with FMD differ from healthy controls, with regards to reaction times or speed of movement, when the supraliminal primes are highly predictable. The same phenomenon might apply to the sense of agency with supraliminal primes. In a future study, an additional condition with a higher cue predictability (95% predictive) could therefore usefully be introduced, and its effect on the sense of agency analyzed.

Thus, the crucial factor that underpins priming effects in FMD, both in terms of motor speed and sense of agency, might be the predictive nature of the prime. A predictive prime typically allows people to strategically prepare their response to the expected imperative stimulus in advance. We suggest that people are normally conscious of such strategic preparation. In FMD patients, this conscious strategic preparation would be maladaptive. In contrast, when primes are non-predictive, they may still facilitate performance through an automatic route, even though they do not allow people to form conscious expectations about the imperative stimulus, or the forthcoming response. Our result suggests that this second, automatic form of motor facilitation functions normally in patients with FMD.

Neurophysiological and neuropsychological studies distinguish two routes to voluntary action. One route, based on the parietal-lateral premotor pathway, predominates in the control of movement in response to sensory stimuli. A second route, based on the prefrontal and medial frontal cortices, predominates in the control of internally-generated, or intentional actions (17, 18). We suggest that the automatic motor facilitation by non-predictive primes, whether subliminal or supraliminal, may be mediated by the first, lateral route. In contrast, strategic motor facilitation, including that provided by predictive primes, might be mediated by the second, medial route. These must remain speculations in the context of the present study, because we did not obtain neurophysiological or neuroimaging data to identify the neural origins of the actions we studied. However, our behavioral findings, together with imaging studies of others (19–22), suggests that the pathophysiology of FMD may involve either dysfunction of this second, medial route, or its inappropriate recruitment. Interestingly, our findings suggest that the lateral route to action remains functional. Thus, therapeutic strategies for FMD might usefully attempt to shift motor control from the medial to the lateral route, and promote motor automaticity rather than motor strategy.

## Data Availability Statement

Our ethics agreement prevents data being openly available, but individual researchers may request anonymized data.

## Ethics Statement

The studies involving human participants were reviewed and approved by NHS Health Research Authority, London–Bromley Research Ethics Committee (reference: 16/LO/1463), and carried out in accordance with the Declaration of Helsinki (23). The patients/participants provided their written informed consent to participate in this study.

## Author Contributions

A-CH designed, conceptualized and programmed the study, recruited the participants, collected, analyzed and interpreted the data, drafted, and revised the manuscript for intellectual content. KB revised the manuscript for intellectual content. ME and PH both designed and conceptualized the study and revised the manuscript for intellectual content. All authors contributed to the article and approved the submitted version.

## Conflict of Interest

The authors declare that the research was conducted in the absence of any commercial or financial relationships that could be construed as a potential conflict of interest.
